# A Case of a Vanished Nose and an Innovative Airway Management Strategy in Post-oncologic Nasal Defect Using Nasopharyngeal Airway

**DOI:** 10.7759/cureus.95001

**Published:** 2025-10-20

**Authors:** Vikash Bansal, Ghazal Ahmed, Abhishek Rai, Ripon Choudhary, Suchita V Meshram, Habib Md R Karim

**Affiliations:** 1 Anaesthesiology, Critical Care, and Pain Medicine, All India Institute of Medical Sciences, Deoghar, Deoghar, IND; 2 Dermatology, Venereology and Leprosy, All India Institute of Medical Sciences, Deoghar, Deoghar, IND; 3 Burn and Plastic Surgery, All India Institute of Medical Sciences, Deoghar, Deoghar, IND; 4 Anaesthesiology, Critical Care, and Pain Medicine, All India Institute of Medical Sciences, Guwahati, Guwahati, IND

**Keywords:** airway challenges, basal-cell carcinoma, nasal destruction, nasal reconstruction, nasopharyngeal airway

## Abstract

Basal cell carcinoma of the nose, when destructive, presents unique reconstructive and airway challenges due to its locally aggressive nature. We report a case of a 68-year-old male who presented with an extensive nasal defect from untreated basal cell carcinoma, requiring complete nasal reconstruction with radial free flap, and flap defect covered with anterolateral thigh graft. Anticipating a difficult airway following flap placement, we implemented a novel strategy: bilateral nasopharyngeal airways were inserted intraoperatively through surgically created openings in the flap. This approach maintained nasal patency, preserved flap integrity, and eliminated the need for a tracheostomy.

The patient had a difficult airway due to limited mouth opening and distorted nasal anatomy. Awake oral fiberoptic intubation was successfully performed. The nasopharyngeal airway was sized to two-thirds of the standard length and secured through the flap with careful suturing to prevent displacement and pressure necrosis. During the postoperative period, humidified oxygen and saline irrigation were employed to avoid crusting and maintain airway hygiene. The nasopharyngeal airways remained in situ for four weeks without complications, until normal nasal passages were established.

This case highlights the importance of multidisciplinary surgery, anesthesia, and airway planning, and introduces a minimally invasive technique that achieves both functional and structural outcomes in complex nasal reconstructions. To our knowledge, prolonged bilateral nasopharyngeal airway use through a free flap has not been previously reported.

## Introduction

Basal cell carcinoma (BCC) is the most common skin cancer worldwide, with approximately 80% of cases occurring on the face, and 25-30% involving the nose. If left untreated, due to its propensity to infiltrate adjacent structures, nasal BCC is classified as high‑risk [1]. Locally aggressive BCC in the nasal region can destroy the nasal septum, lateral wall, and even extend to deeper musculoskeletal structures. Surgical excision with histologically clear margins is the mainstay of treatment. However, reconstruction of extensive nasal defects demands complex free flap techniques, which may compromise airway patency.

Maintaining nasal airway patency after reconstruction is essential-not only for respiration but also for preserving the contour and viability of the grafted flap. Nasopharyngeal airways (NPAs), typically used for short-term airway support in anesthetized or semiconscious patients, are rarely employed for long-term airway maintenance in reconstructive surgery [2,3].

Here, we present a novel use of bilateral NPAs inserted through surgically created tunnels in a anterolateral (radial) forearm flap for total nasal reconstruction after oncologic resection. This method preserved airway patency, eliminated tracheostomy, and supported optimal flap healing.

## Case presentation

A 68-year-old male (weight 64 kg, height 162 cm, BMI 24.38 kg/m²) presented with a large ulcerative lesion over the nasal and upper central maxillary region. The ulcer exposed the nasal cavity and anterior maxilla, with blood-tinged crusting and rolled, pigmented borders. He reported a decade-long history of slow lesion growth, with rapid destruction in the previous year. The patient had been treated empirically for cutaneous tuberculosis without improvement.

**Figure 1:**
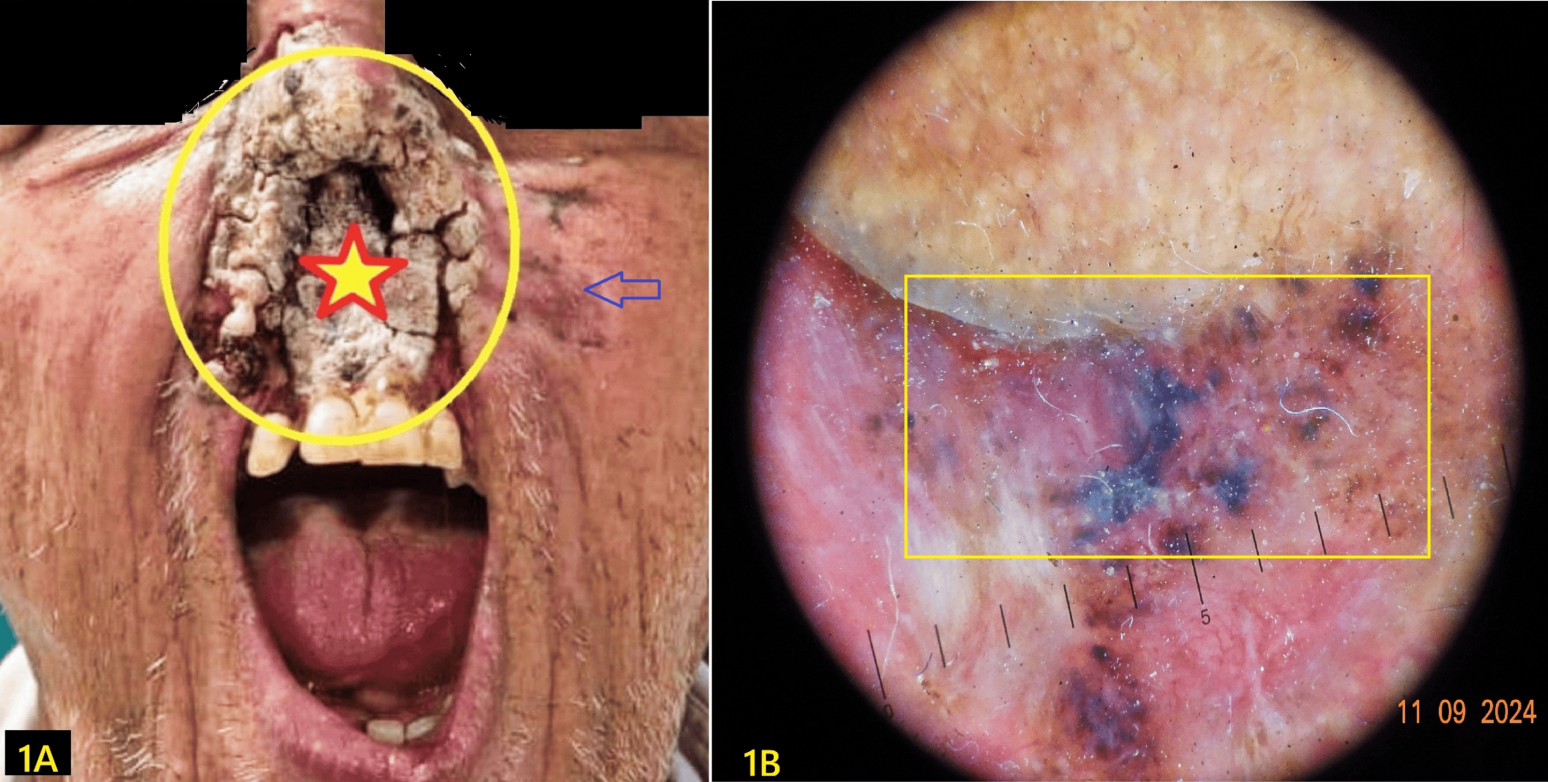
A shows the gross image of destructed upper lip, alae nasi and distal nose with crusted base (star), and erythematous bordering skin (arrow); B is the dermatoscopic image of the bordering skin showing atrophic pigmented border with telangiectasias.

Comorbidities included well-controlled type 2 diabetes mellitus. Dermoscopy revealed telangiectatic vessels and rolled, pigmented borders (Figure 1).

**Figure 2:**
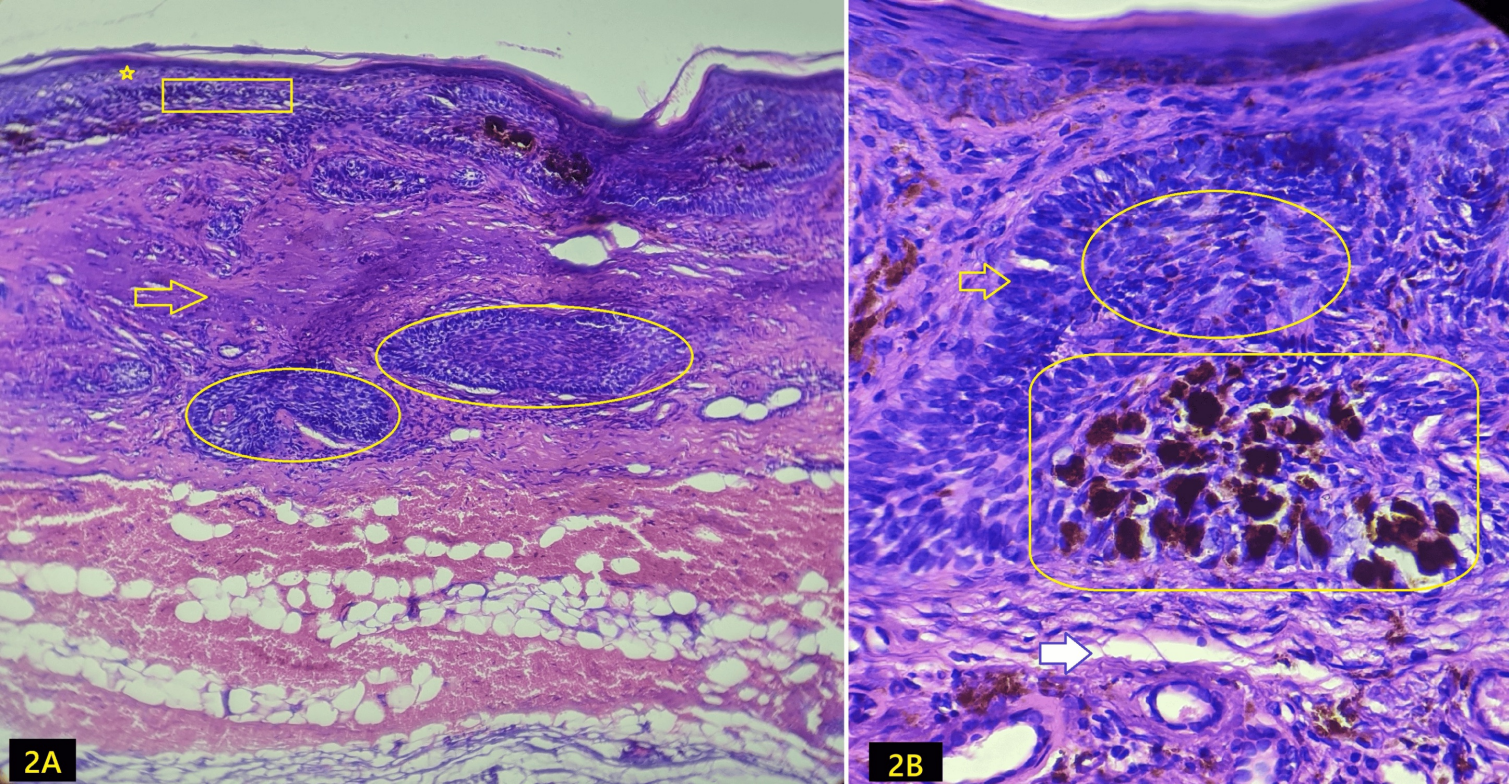
Histopathology (Hematoxylin and Eosin) A- 10x magnification of the lesions showing atrophic epidermis with parakeratosis (star), lichenoid lymphocytic infiltrates (rectangle), solar elastosis (arrow), and nest of basaloid cells (oval) irregular depressed epidermis (arrow), and multiple nests and islands of basaloid cells in dermis; B (40x) shows peripheral palisading (arrow), central haphazard (oval), focal melanin deposits (rectangle) and cleft at stromal-lesion interface (solid white arrow).

Histopathology with Hematoxylin and Eosin stain of the lesions showed that the epidermis is markedly atrophic with focal parakeratosis. The superficial and deep dermis show nests of basaloid cells, with palisading of the cells at the periphery and a haphazard arrangement of those in the center, numerous mitotic figures, and clefts at the stromal-tumor interface, which confirms the diagnosis of nodular BCC (Figure 2). A multidisciplinary team planned a wide local excision with anterolateral (radial) forearm flap reconstruction.

Preanesthetic evaluation revealed vital signs within normal limits: heart rate, 82/min; blood pressure, 130/68 mmHg; SpO₂, 98% on room air; and respiratory rate, 14/min. Airway examination revealed slightly limited mouth opening (~3 cm), modified Mallampati grade 3, protruding teeth, loose lower incisors, and severely distorted nasal anatomy. All these indicated both bag-mask ventilation and nasotracheal intubation, predicting difficult laryngoscopy (Figure 1) [4].

Preoperative labs (Table 1) indicated elevated creatinine with stage 3b chronic kidney disease, and mild anemia. The electrocardiogram showed a normal sinus rhythm; echocardiography demonstrated normal structural integrity, normal valves, an ejection fraction of ~70%, and grade 1 diastolic dysfunction.

**Table 1:** Preoperative laboratory test results with reference values for normal range. HbA1c: glycosylated hemoglobin, eGFR: estimated glomerular filtration rate

Test	Value	Reference values
Haemoglobin	11.7 gm/dl	11-15 gm/dl
Total leucocyte counts	8910/ mm3	4000-11000/mm3
Platelet counts	2.18 lacs /ul	1.5-4.0 lacs/ul
Prothrombin time	12.8 seconds	11-14 seconds
International normalized ratio	1.02	0.8-1.2
Blood sugar (Fasting)	122 mg/dl	60-110 mg/dl
Blood sugar (Post prandial)	159 mg/dl	70-140 mg/dl
Hb1Ac	6.4%	< 6.7%
Blood urea	30.6 mg/dl	10-40 mg/dl
Serum creatinine	2.08 mg/dl	0.2-1.2 mg/dl
eGFR	32 mL/min/1.72 m²	90-120 mL/min/1.72 m²

Awake oral fiberoptic intubation was planned, with tracheostomy on standby. Following nebulization and topical anesthesia, a 7.0 mm endotracheal tube was successfully placed using a fiberoptic bronchoscope and confirmed by waveform capnography [5].

**Figure 3:**
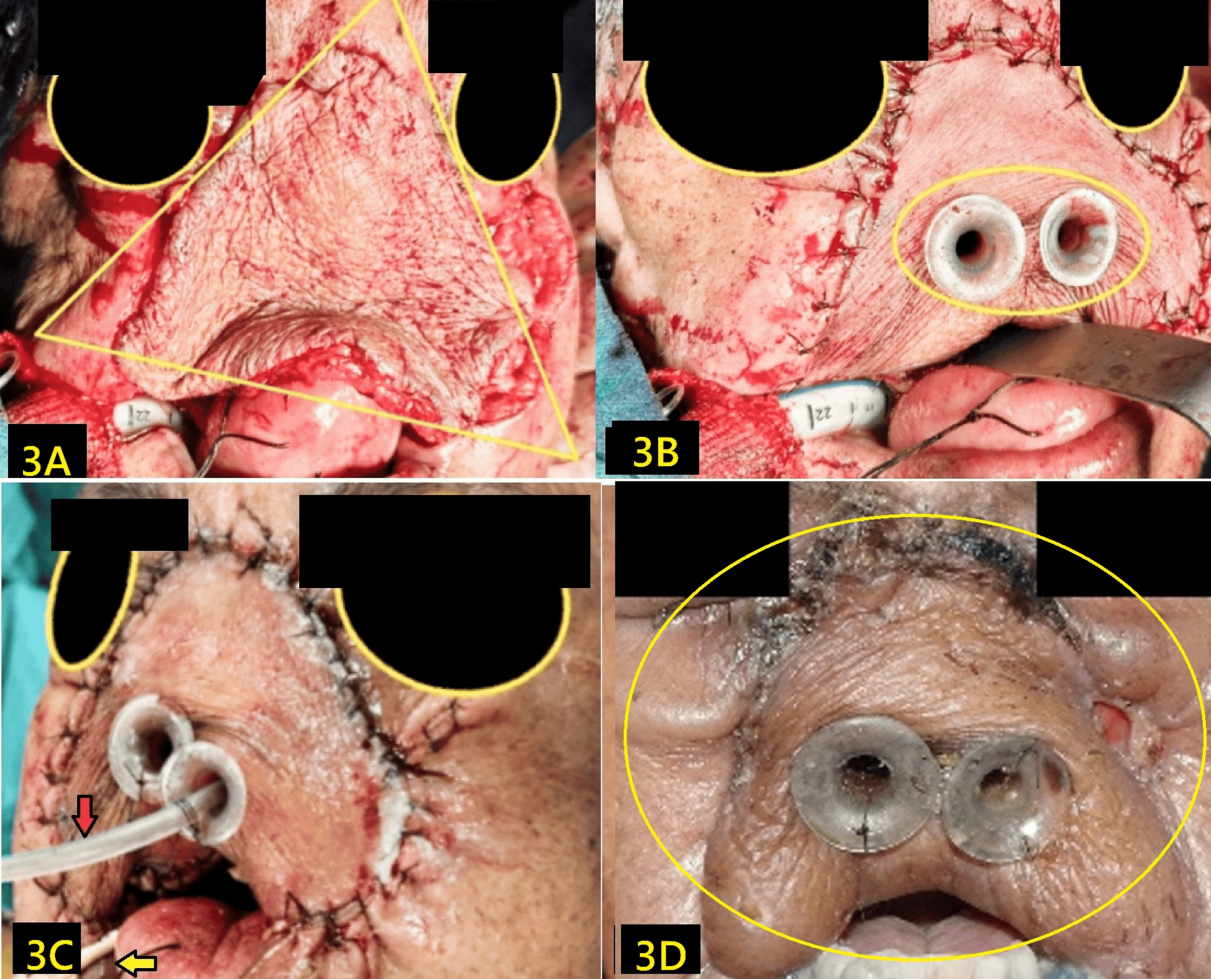
Shows the (radial) forearm flap covering the nasal defect area marked with triangle (A), created nasal openings and NPAs placed marked within circle (B), extubation over airway exchange catheter (horizontal arrow) and Ryle's tube through NPA (vertical red arrow) (C), and postoperative three week image with NPAs are in situ (D). NPA: nasopharyngeal airways

After tumor excision, the radial free flap from the anterolateral aspect was inset to recreate the nasal contour. The flap defect was covered using an anterolateral thigh skin graft. However, due to the flap’s bulk, natural nasal patency was lost entirely (Figure 3A). Anticipating postoperative obstruction, bilateral silicon NPAs were placed intraoperatively through surgically created channels, 3-4 mm in diameter, in the flap. The NPAs were selected at ~ two-thirds the standard length (based on the ala‑tragus distance) (Figure 3B) [6]. They were anchored with absorbable sutures to prevent displacement and pressure necrosis.

After surgery, the patient was safely extubated using an airway exchange catheter (AEC) (Figure 3C). Postoperatively, humidified oxygen and intermittent isotonic saline irrigation were used to prevent mucosal crusting and obstruction. The NPAs were retained for about 28 days, after which the nasal passages returned to normal. No infection, tissue necrosis, or discomfort occurred, and the flap retained good viability and nasal contour (Figure 3D). During the postoperative period, he received an injection of ceftriaxone and metronidazole for five days, followed by tablet linezolid and levofloxacin for another five days. 

## Discussion

Basal cell carcinoma is typically a slow-growing tumor, but it can become locally destructive, especially within high-risk facial areas. The present case shows the aggressive nature of untreated nasal BCC. As it usually presents as a painless small nodule or plaque, or a flesh-colored scaly patch, misdiagnosis or delayed referral, such as empirical anti-tubercular treatment in endemic areas, can worsen prognosis.

Airway management during maxillofacial fractures, dislocations, and nasal reconstruction poses unique challenges when nasal anatomy is severely distorted and surgical access is shared intraoperatively. In many cases, we require techniques other than conventional laryngoscopy [7,8]. In predicted difficult airways, awake fiberoptic intubation remains the gold standard; however, video laryngoscopy also plays a role in selected cases [5,8,9].

Free flap nasal reconstructions can obliterate natural airflow, leading to post-surgical airway compromise. The innovative creation of flap tunnels for bilateral NPA insertion maintained patency, prevented tissue collapse, and preserved nasal architecture [10]. In our case, considering a significant defect, an anterolateral thigh (ALT) graft was initially planned. However, ALT grafts are usually thick, whereas the radial free flap more closely resembles nasal thickness. Further radial free flaps have pliable tissues and long vascular pedicles. We harvested the 16*9 cm^2^ radial free flap from the anterolateral aspect, and the resulting significant defect required a graft harvested from the ALT. The radial artery was anastomosed to the left facial artery, and the cephalic vein to the left common facial vein.

Although prolonged NPA use is rare, potential complications-pressure necrosis, obstruction, and infection-require vigilant observation [11]. In cases similar to ours, prolonged use may even lead to the development of sinusitis. Our patient, however, did ot show any pain, fever, or purulent nasal discharge to indicate clinically significant sinusitis. In this case, humidified oxygen and saline irrigation proved essential for safe long-term use of NPAs and flap success [12]. Nevertheless, an allergic reaction to the device should also be considered. While we have used medical-grade silicon-made NPAs, which usually do not cause severe tissue reactions even when implanted, they show limited perimplantation anti-silicon antibodies [13]. However, delayed hypersensitivity reactions to silicone-made airway devices, such as noninvasive masks, have been reported [14]. Notably, such reactions can even occur with residual sterilization chemicals or concurrently used materials [15].

Nevertheless, type-I reactions can also happen if the patient has a history of previous exposure to silicon and features of allergic reactions [16]. We need to be vigilant and check for skin manifestations, such as erythema, irritation, and dermatitis-like features, during prolonged use. If such reactions are suspected, the device should be replaced with one made of a different material, the dermatitis managed, and, if possible, an allergist consulted. 

This strategy offers a simple, minimally invasive alternative to tracheostomy for selected extensive nasal reconstructions, potentially reducing morbidity. Further studies are needed to confirm its applicability and define patient selection criteria.

## Conclusions

Our case report describes a novel method to ensure airway patency after total nasal reconstruction, using bilateral NPAs inserted through tunnels in the radial free flap. It eliminated the need for tracheostomy, maintained facial contour, and supported flap healing. The method might provide a safe and practical alternative for similar complex reconstructive cases and merits further evaluation.
